# Liquid silicone used for esthetic purposes as a potentiator for occurrence of post-radiotherapy genital lymphedema: case report: Silicone líquido utilizado com finalidade estética como potencializador da ocorrência de linfedema genital pós-radioterapia: relato de caso

**DOI:** 10.1590/1516-3180.2016.0275251016

**Published:** 2017-04-20

**Authors:** Raíssa Quaiatti Antonelli, Davi Reis Calderoni, Igor Ferreira Garcia, Rafael Fantelli Stelini, Adriano Fregonesi, Paulo Kharmandayan

**Affiliations:** I MD. Resident Physician, Department of Surgery, Faculdade de Ciências Médicas da Universidade Estadual de Campinas (FCM/UNICAMP), Campinas (SP), Brazil.; II MD, PhD. Attending Physician. Division of Plastic Surgery, Department of Surgery, Faculty of Medical Sciences, Universidade Estadual de Campinas (UNICAMP), Campinas (SP), Brazil.; III MD. Attending Physician, Department of Pathology, Faculdade de Ciências Médicas da Universidade Estadual de Campinas (FCM/UNICAMP), Campinas (SP), Brazil.; IV MD, PhD. Attending Physician, Division of Urology, Department of Surgery, Faculdade de Ciências Médicas da Universidade Estadual de Campinas (FCM/UNICAMP), Campinas (SP), Brazil.; V MD, PhD. Associate Professor, Head of the Division of Plastic Surgery, Department of Surgery, Faculdade de Ciências Médicas da Universidade Estadual de Campinas (FCM/UNICAMP), Campinas (SP), Brazil.

**Keywords:** Lymphedema, Silicones, Radiotherapy, Reconstructive surgical procedures, Genital diseases, male

## Abstract

**CONTEXT:**

Lymphedema consists of extracellular fluid retention caused by lymphatic obstruction. In chronic forms, fat and fibrous tissue accumulation is observed. Genital lymphedema is a rare condition in developed countries and may have primary or acquired etiology. It generally leads to urinary, sexual and social impairment. Clinical treatment usually has low effectiveness, and surgical resection is frequently indicated.

**CASE REPORT:**

We report a case of a male-to-female transgender patient who was referred for treatment of chronic genital lymphedema. She had a history of pelvic radiotherapy to treat anal cancer and of liquid silicone injections to the buttock and thigh regions for esthetic purposes. Radiological examinations showed signs both of tissue infiltration by liquid silicone and of granulomas, lymphadenopathy and lymphedema. Surgical treatment was performed on the area affected, in which lymphedematous tissue was excised from the scrotum while preserving the penis and testicles, with satisfactory results. Histopathological examination showed alterations compatible with tissue infiltration by exogenous material, along with chronic lymphedema.

**CONCLUSION:**

Genital lymphedema may be caused by an association of lesions due to liquid silicone injections and radiotherapy in the pelvic region. Cancer treatment decisions for patients who previously underwent liquid silicone injection should take this information into account, since it may represent a risk factor for radiotherapy complications.

## RESUMO

**CONTEXTO:**

O linfedema consiste de retenção de fluido extracelular causada por obstrução linfática. Nas formas crônicas, observa-se acúmulo de tecido adiposo e fibrose. O linfedema genital é uma doença rara em países desenvolvidos e pode ter etiologia primária ou adquirida, em geral cursando com disfunções urinária e sexual, bem como com prejuízo do convívio social. O tratamento clínico é, em geral, pouco efetivo, indicando-se com frequência a abordagem cirúrgica, com excisão da área afetada.

**RELATO DE CASO:**

Relata-se o caso de paciente feminina transgênero, encaminhada para tratamento de linfedema genital crônico. Havia antecedente pessoal de tratamento de câncer de canal anal com radioterapia pélvica e de injeções de silicone líquido em glúteos e coxas com finalidade estética. Exames radiológicos mostraram tanto sinais de infiltração tecidual por silicone líquido como granulomas e linfadenopatia como de linfedema. Foi realizado o tratamento cirúrgico da área afetada, com excisão do tecido linfadenomatoso do escroto, preservando o pênis e testículos, com resultado satisfatório. A análise histopatológica mostrou achados compatíveis com infiltração tecidual por material exógeno, bem como com linfedema crônico.

**CONCLUSÃO:**

O linfedema genital pode ser causado pela associação de lesão por injeção de silicone líquido e radioterapia na região pélvica. As decisões no tratamento de neoplasias em pacientes previamente submetidos a injeção de silicone líquido devem levar em conta esse fato, já que pode representar fator de risco para complicações de tratamento radioterápico.

## INTRODUCTION

Lymphedema is a condition of fluid retention in subcutaneous tissue caused by lymphatic obstruction. This implies accumulation of water, macromolecules and proteins in the extracellular space, with impaired influx of leukocytes. Consequently, tissue elasticity is lost, connective tissue deteriorates and infectious processes can can spread more easily. In the chronic phase, lymphedema is also characterized by deposition of fat and fibrous tissue.^1^^,^^2^

In male individuals, this condition may affect the penis or scrotum, or even the entirety of the genital tissue. It is a condition rarely found in developed countries and may have primary etiology (attributed to hypoplasia of the lymphatic vessels) or secondary etiology, as in cases attributed to malignancies, infections, radiotherapy or lymphadenectomy. In tropical regions, it is commonly associated with infections by the parasite *Wuchereria bancrofti* and it has been estimated that up to 20% of men are affected by lymphedema. Idiopathic lymphedema is rare, and is related to genetic disorders such as Milroy’s or Meige’s diseases.^3^^,^^4^ Moreover, genital lymphedema, especially when chronic, affects the patient’s social relations and impairs sexual and urinary functions.

Data from the literature regarding treatments of lymphedema show that conservative therapies (such as use of diuretics, elevation of the affected segment and use of scrotal suspenders) provide poor results, especially in chronic forms with significant fibrosis. In 1820, Delpech described the first surgical technique directed towards resection of lymphedematous tissue and reconstruction using local flaps, which represented a viable option for the treatment of chronic genital lymphedema. Subsequently, novel reconstructive alternatives were developed, with use of posterolateral scrotal flaps, as described by Vaught et al.,^5^ or a combination of scrotal and perineal flaps, as proposed by Halperin et al.^1^

## CASE REPORT

A 36-year-old male-to-female transgender patient was referred to the Plastic Surgery Division with a six-month history of genital lymphedema. She had a past record of AIDS, and had been irregularly treated and followed up at the Infectious Diseases Clinic of our institution for about ten years.

In addition, the patient was attending the Colorectal Surgery Clinic, after being treated for anal canal epidermoid carcinoma. This had been treated non-surgically, with radiotherapy and chemotherapy at the patient’s request and in collaboration with the oncology team. A total of 12 radiotherapy sessions were undertaken, finishing one year prior to presentation to our clinic.

The treatment of the anal cancer was successful, with clinically complete remission of the disease. The patient then presented with complaints of inguinal swelling and pain, along with local edema and erythema, but without fever. A potential relapse of the previous malignancy was suspected and computed tomography scans revealed multiple nodules in the gluteal and thigh regions, some partially calcified, along with bilateral inguinal and obturator lymph node enlargement.

**Figure 1:**
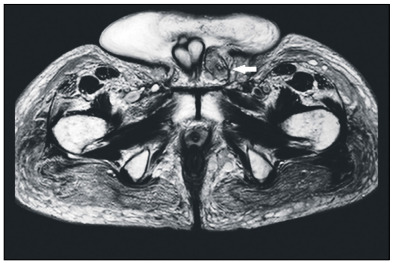
Pelvic magnetic resonance image showing enlargement and infiltration of scrotal soft tissues and presence of nodular images in subcutaneous tissue due to silicone infiltration, such as observed near the base of the penile shaft (arrow).

The condition was further investigated by means of positron emission tomography, which revealed a diffuse hypermetabolic area affecting the subcutaneous tissue around the pelvis, with exogenous material, and extending to the hypogastric, scrotum and proximal thigh regions. This was compatible with an extensive inflammatory process, and a focal hypermetabolic area was observed in the right inguinal region, probably corresponding to a lymph node. Magnetic resonance imaging (Figure 1) showed diffuse subcutaneous edema, more prominently at the scrotum and penis, as well as lipoma-like masses along the left spermatic cord. After the imaging results had been obtained, the patient revealed that she had previously undergone liquid silicone injections to the buttocks and thighs for esthetic purposes.

**Figure 2:**
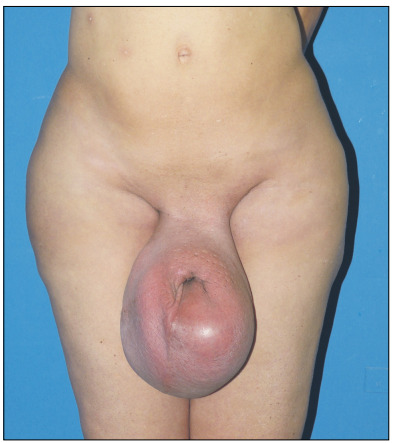
Preoperative appearance of the patient showing massive scrotal enlargement with edematous and erythematous skin and loss of penile shaft contour.

As there was no sign of recurrent malignancy, the patient was referred for plastic surgery and urology consultations. The initial physical examination showed significant enlargement of the scrotum, with erythematous tender infiltrated skin. The testicles were not palpable and there was loss of penile shaft contour, with no visible transition between its ventral surface and the scrotum (Figure 2).

After multispecialty discussion, it was decided with the patient’s consent to excise all the affected scrotal tissue while preserving the penis and testicles, and to reconstruct the perineal defect with skin grafts or local thigh flaps. The patient did not desire scrotal reconstruction.

**Figure 3:**
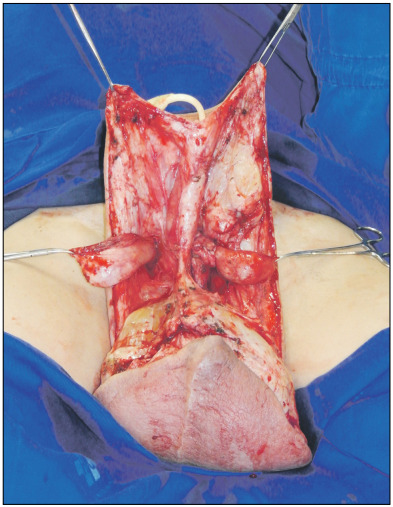
Intraoperative view of the lymphedematous tissue dissection, preserving both testicles, and the lateral skin flap design used for reconstruction.

The surgical procedure was performed jointly by the plastic surgery and urology teams, starting with dissection of the testicles and spermatic cords (Figure 3). Identification and excision of a hard mass from the left cord was performed, and this mass was later shown through pathological analysis to be compatible with chronic lymphedema and stromal alterations relating to previous radiotherapy.

**Figure 4:**
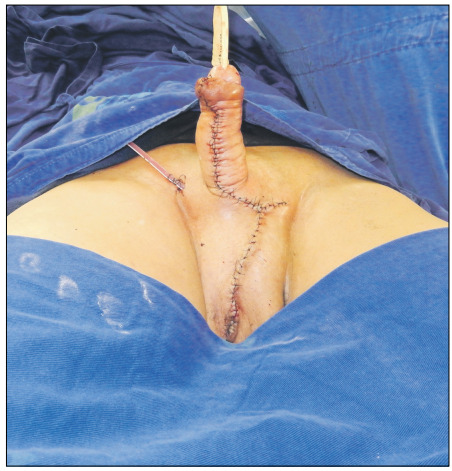
Immediate postoperative result.

Subsequently, the penile shaft was separated from the scrotal tissue, while preserving lateral unaffected skin flaps. The product of scrotal tissue excision weighed 1,430 g and was also sent for pathological examination. There was no need for skin grafts or thigh flaps for reconstruction, since the remaining lateral skin permitted primary closure of the wound without scrotal reconstruction, as desired by the patient (Figure 4).

**Figure 5:**
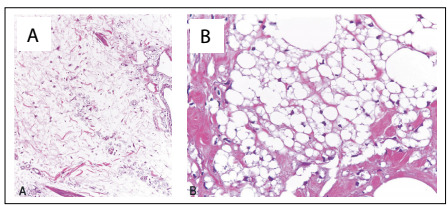
A: marked stromal edema with scattered macrophages containing intracytoplasmic clear vacuoles (100 x, hematoxylin and eosin, HE); B: extracellular empty pseudomicrocysts (top and bottom right) and numerous macrophages with intracytoplasmic clear vacuoles of different sizes, sometimes scalloping the nucleus and thus providing an appearance similar to a lipoblast (400 x, HE).

The histopathological analysis on the resected tissue revealed signs of chronic lymphedema, and presence of frequent lipoblast-like multivacuolated cells, which could be explained by the presence of exogenous material such as liquid silicone phagocytized by macrophages (Figure 5). Immunohistochemical analysis revealed that these cells were positive for CD68 and negative for S100 protein. There were also areas with extracellular empty pseudocysts of different sizes and scattered perivascular and interstitial inflammatory infiltrate, predominantly lymphomononuclear. Special stains did not show any mycobacteria or fungus.

**Figure 6:**
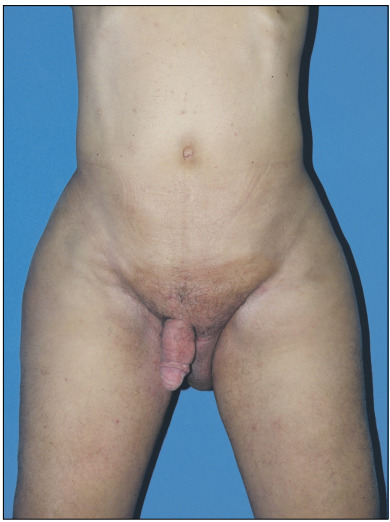
Postoperative result after a 10-month follow-up with no lymphedema relapse.

The postoperative period was mostly uneventful. The patient presented a single complication consisting of minor dehiscence of the superficial operative wound in the ventral portion of the penis, which was managed with dressings. The final result was considered satisfactory by both the patient and the surgical team, with no signs of lymphedema relapse after nearly one year of follow-up (Figure 6).

## DISCUSSION

Genital lymphedema is a disorder that implies complex treatment, especially in its chronic form with tissue fibrosis. It is a debilitating and psychologically stressing condition.^1^^,^^6^^,^^7^^,^^8^^,^^9^^,^^10^ In the present case, the patient presented acquired scrotal lymphedema, probably caused by radiotherapy. The presence of liquid silicone in the subcutaneous tissue and pelvic lymph nodes was also a contributory factor.

Some cases of scrotal lymphedema associated with radiotherapy are presented in the literature, and there is a single case of lower-limb lymphedema in a patient who previously underwent liquid silicone injection to the gluteal and thigh regions for esthetic purposes (Table 1).^11^ However, to date, there has been no report of these two factors occurring together as a cause of lymphedema, and our case is thus the first one.

**Table 1:**
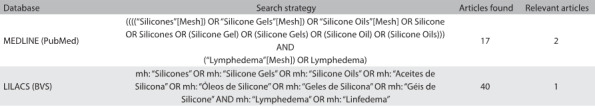
Search strategies used for electronic databases

It could be postulated that radiotherapy tissue damage acted as a precipitating factor for dysfunction in a lymph drainage system that was already impaired by the presence of liquid silicone. Histopathological findings compatible with infiltration of the scrotal tissue by liquid silicone corroborate the hypothesis that the material would be a contributory factor for development of lymphedema. In the previous description, both the regional lymphadenopathy and the presence of silicone granulomas, which exert vascular extrinsic compression, were reported to be causative mechanisms for compromised lymphatic drainage.^11^ Furthermore, in another report, patients who underwent injections in the pelvic area were described as presenting symptoms such as lower-extremity edema and varices, which were attributed to inguinal adenopathy and vascular compression due to local fibrosis.^12^

Although the initial studies demonstrated that injectable silicone is biologically inert, subsequent reports have shown the presence of inflammatory reaction associated with its use as filler.^13^ It has been discussed in the literature whether this reaction would take place against the silicone itself, or against its degradation products, or against impurities in or contamination of the substance applied.^13^ Histological findings associated with liquid silicone application may include extracellular empty pseudocysts of different sizes, reminiscent of a “Swiss cheese pattern”, and macrophages with intracytoplasmic clear vacuoles, sometimes indentating the nucleus and providing an appearance similar to a lipoblast. This last finding may lead to misdiagnosis of liposarcoma, since lipoblasts are often found in this malignancy. Clinical-pathological correlation is important, since this provides information on the history and site of silicone injection.^14^ Immunohistochemically, it is expected that pseudolipoblasts present in the silicone foreign body reaction will be negative for S100 protein, unlike many liposarcomas, which present lipoblasts that are positive for this marker. Our patient was indeed S100-negative.

The results from the surgical procedure were deemed satisfactory. Local reconstruction was achieved by means of primary closure in a single operation, without erectile dysfunction or impairment of penile sensitivity. Because the patient is male-to-female transgender, she considered that absence of a scrotal sac was a positive outcome that was more appropriate for her gender identity.

## CONCLUSION

The presence of liquid silicone injected for esthetic purposes may represent a risk factor for development of lymphedema in patients who undergo radiotherapy at the same anatomical site, thus potentiating the occurrence of this rare complication.
